# Sarcopenia, obesity, and their association with selected behavioral factors in active older adults

**DOI:** 10.3389/fphys.2023.1129034

**Published:** 2023-02-23

**Authors:** Kaja Teraž, Miloš Kalc, Manca Peskar, Saša Pišot, Boštjan Šimunič, Rado Pišot, Primož Pori

**Affiliations:** ^1^ Institute for Kinesiology Research, Science and Research Centre, Koper, Slovenia; ^2^ Faculty of Sport, University of Ljubljana, Ljubljana, Slovenia; ^3^ Faculty of Medicine, University of Maribor, Maribor, Slovenia; ^4^ Biological Psychology and Neuroergonomics, Department of Psychology and Ergonomics, Faculty V: Mechanical Engineering and Transport Systems, Technische Universität Berlin, Berlin, Germany

**Keywords:** aging, physical activity, nutrition, healthy lifestyle, body composition

## Abstract

**Introduction:** The number of obese people in the world is increasing, as is the number of sarcopenic people among the older adults. Although both states are concerning, they can be positively influenced by selected behavioral factors such as adequate nutrition and physical activity. We were interested in the prevalence of sarcopenic obesity in active older people and the influence of behavioral factors on this phenomenon.

**Methods:** The study included 38 older adults (21 women) with a mean age of 75.3 ± 5.0 years. Sarcopenic parameters were determined with different tests: Handgrip Test, Chair Stand Test, Gait Speed, Timed Up and Go Test, and Short Physical Performance Battery. Body composition was measured by dual-energy x-ray absorptiometry. Physical activity level was measured using accelerometers, and nutritional status was assessed using the Mini-Nutritional Assessment and MEDLIFE Index questionnaire.

**Results:** Of all included active participants (the average number of steps per day was 8,916 ± 3,543), 47.4% of them were obese. Of all included women, 52.4% were obese. Sarcopenic obesity was found in three (7.9%) participants. Nutritional status correlated with strength of lower extremities and physical performance tests (gait speed, Timed Up and Go Test and Short Physical performance battery). Higher number of steps per day positively correlates with physical performance.

**Discussion:** Interestingly, we did not find any correlation between the main obesity parameter such as percent body fat or body mass index (and thus sarcopenic obesity) and any of the selected behavioral factors (physical activity, sedentary behavior, or dietary habits). In conclusion, reaching the recommended levels of physical activity in older adults may not be sufficient to prevent the occurrence of obesity and sarcopenic obesity.

## 1 Introduction

The population is ageing, but there is little evidence that living longer life is accompanied by healthier ageing (Beard et al., 2016). As the population ages, the number of diseases directly related to behavioral factors increases (Kruger et al., 2009), these include obesity and degenerative and involuntary skeletal muscle disorders, also known as sarcopenia. In Europe, 21.2% of older adults aged 65–74 years were obese in 2017 (European Commission. Statistical Office of the European Union. 2020), this number rises to 22.3% in 2019 (European Union, 2022). Similar is expected to be true for sarcopenia as well; it is expected the increase of sarcopenia from 11.1% in 2016 to 12.9% in 2045 (Ethgen et al., 2017). Moreover, sarcopenia and obesity often coexist and the prevalence of both independent mortality factors (obesity and sarcopenia) has increased in older adults in recent years (World Health Organization, 2000; Cruz-Jentoft et al., 2010; Cruz-Jentoft et al., 2014). The combination of them leads to a specific condition named sarcopenic obesity (Roubenoff, 2004). Namely, a decline in muscle mass and a gain in fat mass (both visceral fat and intramuscular fat) often coincide and can lead to sarcopenic obesity (SO) (Gallagher et al., 2000). According to the European Working group on Sarcopenia in Older People 2 (EWGOSP2) (Cruz-Jentoft Alfonso et al., 2019), the definition of sarcopenic obesity is a condition of a reduced lean body mass in the context of excess adiposity. The lack of consensus on its definition persisted till this year when The European Society for Clinical Nutrition and Metabolism (ESPEN) and the European Association for the Study of Obesity (EASO) reached a consensus on a definition (Donini et al., 2022).

Among the most important factors that can contribute to the faster progression of sarcopenia, obesity and sarcopenic obesity are behavioral factors, e.g., inadequate dietary habits and insufficient physical activity (PA) with higher levels of sedentary behavior. The beneficial effect of PA in general for the prevention of sarcopenia has been already established (Steffl et al., 2017). In addition, the positive effects of adequate nutrition on muscle mass maintenance have been repeatedly reported in the literature (Marcos-Pardo et al., 2020). In addition, with proper exercise habits and optimal nutrition, we can have a positive impact on the occurrence of obesity and morbid obesity (Marcos-Pardo et al., 2020; Zhuang et al., 2022). The Mediterranean diet is an example of a dietary pattern that illustrates the relationship between dietary quality and healthy ageing (Milte and McNaughton, 2016). The Mediterranean diet and lifestyle, includes a diet high in nutrients, whole grains, vegetables, fruits, fish, olive oil, and herbs combined with a certain lifestyle such as regular exercise, social interaction, and so on. Health benefits of the Mediterranean diet and Mediterranean lifestyle have been already well established; adherence to a Mediterranean lifestyle and thus also diet is associated with a more than 50% lower rate of all-cause and cause-specific mortality (Diolintzi et al., 2019), while higher adherence to the Mediterranean diet is specifically associated with lower odds of sarcopenia among older adults (Hashemi et al., 2015). An important part of the Mediterranean lifestyle is also regular physical activity. Current recommendations for healthy older adults encourage at least 150 min of aerobic physical activity of moderate-to-vigorous intensity per week to maintain functional abilities (Bull et al., 2020). In addition, muscle and bone strengthening activities that activate major muscle groups should be performed at least two times a week (Tremblay et al., 2011; Bull et al., 2020).

As far as we know, no study to this day investigated the association of sarcopenia and obesity in active older adulty in connection with behavioral factors such as physical activity and nutritional assessment. The aim of this study was to evaluate the presence of sarcopenia and obesity parameters in selected active older adults and to determine whether behavioral factors such as the amount of physical activity, sedentary behavior, nutritional status, and Mediterranean lifestyle show an association with it.

## 2 Materials and methods

### 2.1 Participants

The current study is a cross-sectional analysis of active older adults, aged above 65 years, who previously participated in the Physical Activity and Nutrition for Great Aging (PANGeA) mass measurements study in 2013 and were again invited for follow-up measurements in 2021. For the purpose of this study, we were interested in 52 active older adults, aged between 65 and 85 (22 men and 30 women, mean age: 68.4 ± 5.6 years), who participated in follow-up measurements in 2021. Participants were from Slovenian coastal area (Koper and surroundings) which applies to the Mediterranean (Adriatic) part of Slovenia. Participants were invited to the measurements *via* mail and additionally *via* phone call. Measurements included anthropometric and body composition measurements, motor tests, and different questionnaires such as information about socio-demographic data, information on health status, drug therapy and The Montreal Cognitive Assessment (MoCA) (Nasreddine et al., 2005) screening tool for cognitive impairment was used to obtain a measure of general cognitive functioning.

The study was conducted in accordance with the Declaration of Helsinki and approved by the National Ethical Committee of the Slovenian Ministry of Health (ethical approval No. 0120-76/2021/6) and confirmed by the ZRS Koper Scientific Council No. 0624–77/21. Moreover, the clinical trial protocol was registered on ClinicalTrials.gov, Identifier: NCT04899531. Informed consent was obtained from all participants involved in the study.

### 2.2 Measurements

#### 2.2.1 Anthropometric characteristics and body composition

During the measurement of anthropometric characteristics and body composition, participants wore only light underwear and no shoes. Body mass (BM) was measured to the nearest 0.1 kg using a hand scale (Seca 709, Hamburg, Germany), and height was measured to the nearest 0.5 cm using a standardized wall-mounted height board. Body mass index (BMI) was calculated as the participant’s body mass in kilograms divided by the square of body height in meters.

Body composition (total mass, appendicular fat, skeletal muscle mass, and bone mass) was measured with a dual-x-ray absorptiometry (DXA) (Lunar Prodigy; GE Medicals, Madison WI). The DXA was calibrated daily with a standard phantom.

#### 2.2.2 Sarcopenia parameters

Sarcopenia parameters were assessed using two tests of physical performance suitable for the age group and DXA analysis as described above. A representative spectrum of the target group’s physical abilities was thus obtained. All cut-off points for sarcopenia parameters [skeletal muscle mass, hand grip strength, Chair stand test, gait speed, Timed-Up and Go test (TUG) and Short physical performance battery (SPPB)] were identified using algorithm of the EWGSOP2 (Cruz-Jentoft Alfonso et al., 2019).

##### 2.2.2.1 Muscle strength

Skeletal muscle strength was evaluated by two tests: The hand-grip test for evaluating muscle strength of the upper extremities and Chair stand test for evaluating muscle strength of lower extremities. The hand-grip test was evaluated with a hand dynamometer (Yamar, Patterson Medical, United Kingdom). The participant performed the test with dominant hand in a seating position with the elbow flexed at 90° and positioned on the side, but not against, the trunk. The hand was positioned firmly on the dynamometer with the thumb pointing up. The average of three trails measured in kilograms was considered for further analysis. Lower extremity muscle strength was measured using the Chair stand test. It was performed in accordance with established protocol (Ng et al., 2015). Shortly, participants were instructed to stand up, from a seated position, and sit down as quickly as possible. After five repetitions, the administrator noted the time required. Performance impairment on the Chair stand was defined using the EWGSOP210 cut-off >15 s.

##### 2.2.2.2 Muscle mass

Appendicular skeletal muscle mass (ASM) was evaluated using DXA (see Anthropometric characteristics and body composition section). Moreover, the absolute level of ASM was adjusted for body height squared (ASM/height^2^) to obtain skeletal muscle index (SMI) (Shepherd, 2016).

##### 2.2.2.3 Physical performance

Physical performance was evaluated by determining gait speed, Timed up and go test (TUG), and the Short Physical Performance Battery (SPPB). To evaluate gait speed the participant walked for 1 min and 30 s in their usual pace between 4 m long system, from one side to another. Slow gait speed was defined using EWGSOP2(Cruz-Jentoft Alfonso et al., 2019) reference values of ≤0.8 m/s. The sum of mobility, balance, and ability to perform activities of daily living was measured with a validated TUG test (Beauchet et al., 2011). The time it took participants to stand up from a chair, walk a distance of 3 m, turn around, return to the chair, and sit down again (in seconds) was measured. The time was measured with a hand-held stopwatch (Podsiadlo and Richardson, 1991). Slow TUG performance was defined using EWGSOP2 (Cruz-Jentoft Alfonso et al., 2019) reference value of ≥20 s. *The SPPB* is an assessment that includes five tests for lower limb function including balance, strength, gait and endurance and it is used for characterization of lower extremity function (Guralnik et al., 1994). Three individual measures of physical performance include gait speed, balance test and Chair stand test. The aim of the balance tests was to stand for 10 s with the feet together in side-by-side, semi-tandem, and tandem positions and unaided, with the test progressing in difficulty after successful completion. Gait speed was assessed as previously described (see “gait speed”), and the Chair stand test was performed as described above (see “Chair stand test”). The total SPPB score ranging from 0 to 12; higher scores indicate better performance (Kwon et al., 2009).

#### 2.2.3 Physical activity level and sedentary behavior

The amount and intensity of physical activity and sedentary behaviour was assessed using an Actigraphy GT3x accelerometer (Actigraph, Pensacola, FL, United States). We obtained data on the amount of moderate to vigorous physical activity (MVPA), light physical activity (LPA), and sedentary behavior (SB). Participants were instructed to wear an accelerometer around their waist for seven consecutive days (five weekdays and two weekend days) during all waking hours. Participants were instructed to put on the accelerometer in the morning after waking and to wear it until bedtime. The exception was water activities (e.g., showering, swimming), in which case they had to remove the accelerometer and put it back on, after the activity. Each day, they recorded when the accelerometer was installed and when it was stored in the attached logbook. The recorded data was then used to calculate the valid minutes the accelerometer was worn. Accelerometers were preprogrammed to save data in 10 s epochs and attached with an elastic strap on their right hip. Data were processed using standard methods; raw data were converted to counts per minute (cpm), which reflects the acceleration and hence the intensity of PA, data of 20 min of consecutive zeros were removed. Inclusion criteria for data validation were at least 10 h (>600 min) (Choi et al., 2011; Hart et al., 2011) of wearing time for a valid day and at least 5 days for a valid record, including one weekend day (Mâsse et al., 2005; Trost, Mciver, and Pate, 2005). From a valid record, an overall physical activity on cpm was calculated. The higher the cpm, the higher intensity of movement measured. PA intensity is typically categorized based on metabolic rate (RMR). Each valid minute of wearing time was divided into three intensity categories. Cut-off points were determined by converting vector accelerometer magnitude from cpm and corresponded to previously standardized MET values: SB (<1.5 MET in supine or seated position), LPA (1.5–2.99 MET), and MVPA (>3 MET). We used cut-off that are typical for older adults. Time spent in MVPA was determined based on the established accelerometer count cut-point of >1,041 cpm.

#### 2.2.4 Nutritional status and mediterranean lifestyle

Nutrition status was evaluated using Mini Nutritional Assessment (MNA) (Guigoz and Vellas, 1997). The questionnaire is an internationally validated tool to assess the risk of malnutrition and nutritional status. The questionnaire can be scored from 0 to 30 points, interpreted as well nourished (≥24 points), at risk of malnutrition (17–23.5 points), or undernourished (<17 points).

The Mediterranean lifestyle was assessed using The Mediterranean lifestyle (MEDLIFE) index (Sotos-Prieto et al., 2015). The MEDLIFE index consists of 28 questions divided into three groups. The groups include food consumption, traditional dietary habits, and physical activity, rest and social contacts. The participant can score 1 or 0 points for each question, giving a maximum total of 28 points for the entire questionnaire. The more points, the more one adheres to the Mediterranean lifestyle.

#### 2.2.5 Obesity

Following the example of the study by Bahat et al. (2020) and Bahat (2022) obesity was defined by two different methods; if a participant had a fat percentage (FM) above the 60th percentile [the Zoico method (Zoico et al., 2004)] or BMI of a participant was of ≥30 kg/m^2^ [the WHO definition of obesity (World Health Organization 2010)]. The obesity cut-off according to Zoico’s definition was 29.1% FM for men and 35.8% FM for women.

#### 2.2.6 Sarcopenia

Sarcopenia was defined in three stages: probable sarcopenia, sarcopenia and severe sarcopenia. Probable sarcopenia was confirmed if low muscle strength was identified (grip strength lower than 27 kg for men and 16 kg for women and/or chair stand test was completed in more than 15 s for five rises), sarcopenia was confirmed if low muscle strength was noted in addition to SMI lower than 7.0 kg/m^2^ for men and 5.5 kg/m^2^ for women was identified, severe sarcopenia was confirmed when participants were diagnosed with low muscle strength and low physical performance measured with gait speed and/or TUG and/or SPPB (gait speed cut-off ≤0.8 m/s, TUG cut-off ≥20 s, SPPB cut-off ≤8 point score).

#### 2.2.7 Sarcopenic obesity

As proposed by Donini et al. (2022), we established a two-step process for defining sarcopenic obesity: screening and diagnosis. The screening process involved identifying participants who had increased BMI (≥30 kg/m^2^) and were 70 of age or older, along different clinical symptoms or health risk factors, listed by Donini et al. (2022). Once screening was confirmed, the diagnosis followed: sarcopenic obesity was diagnosed when obesity and sarcopenia coexisted in the participant.

### 2.3 Statistical analysis

All anthropometric characteristics, and sarcopenic parameters are described using mean and standard deviation (SD) The normality of the distribution was checked statistically (the Shapiro-Wilk test) and then confirmed graphically (histogram and QQ plots), which was implemented to evaluate their normal distribution. Comparison of selected sarcopenia and obesity parameters between sexes were made with independent-samples *t*-test. The correlation between sarcopenia and obesity parameters (lower extremities strength, BMI, gait speed, TUG and SPPB) and selected behavioral variables was evaluated using Pearson’s r correlation coefficient. Due to differences between sexes, non-parametric Spearman’s rank correlation coefficients was used for SMI, handgrip strength, FM (%) and selected behavioral variables. Multiple regression analysis was used to analyze all sarcopenia obesity parameters; hand grip strength, lower extremities strength, SMI, FM (%), BMI, gait speed, TUG, and SPPB. The covariates included in the models were age, amount of MVPA and SB, number of steps per day, adherence to the Mediterranean lifestyle, and nutritional status. All statistical analyses were performed by IBM SPSS Statistics 22 (SAS Institute, Cary, NC, United States), with a significance level set at *p* < .05. G*Power (Faul et al., 2009) was used for effect size calculation, using Cohen’s d (*α* = 0.05, power = 0.95).

## 3 Results

Of the 52 enrolled participants, 38 were included in the analysis, and 14 were excluded due to refusal to wear an accelerometer (*N* = 5) or did not meet the inclusion criteria for data validation of accelerometer (*N* = 3), six of participants did not perform body composition measurements due to difficulty lying on their back. Socio-demographic characteristics of the sample are presented in Table 1. Application of the chosen definition of sarcopenia, obesity, and sarcopenic obesity indicated that a total of three participants were sarcopenic (two women), 18 participants were obese (61.1% were women) and three participants had sarcopenic obesity (two women).

**TABLE 1:** General characteristics of the study participants.

	Total *n* = 38	Men *n* = 17	Women *n* = 21
Age (y)	75.3 ± 5.2	74.4 ± 4.9	76.1 ± 5.2
Education			
Primary	—	—	—
Secondary, *n* (%)	18 (47.4)	8 (47.1)	10 (47.6)
Collage/University, *n* (%)	20 (52.6)	9 (52.9)	11 (52.4)
Marital status			
Married, *n* (%)	25 (65.8)	14 (82.4)	11 (52.4)
Other, *n* (%)	13 (34.2)	3 (17.6)	10 (47.6)
Status of living			
Alone, *n* (%)	13 (34.2)	1 (5.9)	12 (57.1)
In company, *n* (%)	25 (65.8)	16 (94.1)	9 (42.9)
Number of medications	1.8 ± 1.7	2.1 ± 1.6	1.6 ± 1.8
Number of comorbidities	3.5 ± 2.2	3.1 ± 1.8	3.7 ± 2.4
MoCA	25.2 ± 3.2	24.8 ± 3.1	25.6 ± 3.2
Sarcopenia, *n* (%)	3 (7.9)	1 (5.9)	2 (9.5)
Obesity, *n* (%)	18 (47.4)	7 (41.2)	11 (52.4)
Sarcopenic obesity, *n* (%)	3 (7.9)	1 (5.9)	2 (9.5)

^a^ MoCA, Montreal cognitive assessment.

There was a statistical difference between women and men (Table 2) in body mass (*t* = 4.452; *p* < .001; Cohen’s *d* = 1.47), body heights (*t* = 9.868; *p* < .001; Cohen’s *d* = 3.18), fat-free mass expressed in kilograms (*t* = 9.696; *p* < .001; Cohen’s *d* = 3.11) and in percentages (*t* = 3.082; *p* = .004; Cohen’s *d* = 0.97), fat mass (*t* = −3.407; *p* = .002; Cohen’s *d* = 1.08), SMI (*t* = 4.930; *p* < .001; Cohen’s *d* = 1.56) and hand grip strength (*t* = 7.890; *p* < .001; Cohen’s *d* = 2.65). We did not find any statistical difference in selected behavioral factors (MVPA, SB, steps, MNA, and MEDLIFE index) between the sexes.

**TABLE 2:** Sarcopenia and obesity parameters between sexes.

	Total	Men	Women	*p (Cohen d)*
N	38	17	21	N.A.
Body mass (kg)	72.4 ± 15.2	82.3 ± 10.6	64.4 ± 13.6	<.001 (1.47)
Body height (cm)	167.2 ± 9.1	175.7 ± 5.1	160.4 ± 4.5	<.001 (3.18)
Body mass index (kg/m^2^)	25.7 ± 4.2	26.6 ± 2.8	25.0 ± 5.1	.247
Fat free mass (kg)	49.2 ± 10.4	58.9 ± 6.4	41.3 ± 4.8	<.001 (3.11)
Fat free mass (%)	68.3 ± 7.4	71.9 ± 4.9	65.5 ± 7.9	.004 (0.97)
Fat mass (%)	31.8 ± 7.1	28.1 ± 4.4	34.8 ± 7.6	.002 (1.08)
Skeletal muscle index (kg/m^2^)	7.1 ± 1.2	7.9 ± 0.9	6.5 ± 0.9	<.001 (1.56)
Hand grip strength (kg)	32.8 ± 12.1	43.6 ± 9.0	24.1 ± 5.2	<.001 (2.65)
Chair Stand test (sec)	9.6 ± 2.8	10.1 ± 2.7	9.3 ± 2.9	.369
Gait Speed (m/s)	1.1 ± 0.2	1.1 ± 0.3	1.1 ± 0.2	.635
TUG (sec)	6.7 ± 1.4	6.8 ± 1.7	6.6 ± 1.0	.613
SPPB	11.4 ± 0.9	11.4 ± 0.8	11.5 ± 1.1	.834
AWT/day	913.7 ± 102.4	936.3 ± 97.9	895.3 ± 104.6	.224
MVPA (min/day)	98.0 ± 44.6	95.6 ± 39.6	99.9 ± 49.1	.769
SB (min/day)	646.8 ± 87.2	673.7 ± 84.4	625.1 ± 85.2	.336
Steps/day	8,916 ± 3,543	9,007 ± 3,700	8,842 ± 3,500	.889
MNA	27.4 ± 1.9	27.6 ± 1.9	27.2 ± 1.9	.581
MEDLIFE	18.2 ± 3.3	18.0 ± 3.7	18.3 ± 3.0	.794

^a^ TUG, Timed Up and Go test; SPPB, Short Physical Performance Battery; AWT, accelerometer wearing time; MVPA, moderate to vigorous physical activity; SB, sedentary behavior; MNA, mini nutritional assessment.

Table 3 shows that handgrip strength and SMI are moderately related to age (negative correlation). Lower extremity strength was moderately related to the nutritional status (both negatively correlated). In the physical performance tests, all selected parameters showed a relationship with selected behavioral variables; gait speed had a moderate relationship with the number of steps and nutritional status (positive correlation), TUG had a moderate relationship MVPA and number of steps (both negative correlation) and nutritional status (positive correlation), SPPB had a moderate positive correlation with nutritional status.

**TABLE 3:** Pearson’s and Spearman’s (*) correlation between sarcopenia and obesity parameters and selected behavioral variables. A Pearson’s r and Spearman’s rho (*p*-value) is reported.

Variables	HGS*	CHT	SMI*	FM*	BMI	GS	TUG	SPPB
Age (y)	−.351 (.031)	.136 (.416)	−.324 (.047)	.212 (.202)	−.051 (.762)	−.231 (.163)	.239 (.148)	−.264 (.110)
MVPA (min)	.155 (.354)	−.161 (.333)	.028 (.868)	−.123 (.461)	−.182 (.275)	.161 (.334)	−.371 (.022)	−.034 (.840)
SB (min)	−.002 (.989)	.045 (.786)	.157 (.346)	−.175 (.294)	.100 (.550)	−.056 (.739)	.065 (.697)	.039 (.816)
Steps	.207 (.257)	−.313 (.056)	−.001 (.995)	.058 (.754)	−.221 (.182)	.333 (.041)	−.391 (.015)	.153 (.360)
MNA	.141 (.398)	−.441 (.006)	.243 (.142)	.131 (.434)	.099 (.553)	.352 (.030)	.454 (.004)	.379 (.019)
MEDLIFE index	−.183 (.272)	.081 (.630)	−.209 (.208)	.050 (.767)	−.132 (.428)	−.224 (.175)	−.053 (.754)	.119 (.477)

^a^HGS, hand grip strength; CHT, chair stand test; SMI, skeletal muscle index; FM, fat mass; BMI, body mass index; GS, gait speed; TUG, Timed Up and Go test; SPPB, Short Physical Performance Battery; MVPA, moderate to vigorous physical activity; SB, sedentary behavior; MNA, mini nutritional assessment.

Multiple regression was run to predict different sarcopenia and obesity parameters such as age, physical activity, number of daily steps and nutritional status (Table 4).

**TABLE 4:** Multiple linear regression analysis of the effect of age, physical activity, number of daily steps and nutritional status on sarcopenia parameters.

	B	*β*		SE	*p*	Partial *r*	VIF
Hand grip strength	R = .367; F (1,36) = 5.612; *p* = .023						
Constant	99.188			28.095	.001		
Age	−.882	−.367		.372	.023	−.367	1.000
Chair stand test	R = .441; F (1,36) = 8.715; *p* = .006						
Constant	27.708			6.134	<.001		
MNA	−.660	−.441		.224	.006	−.441	1.000
SMI	R = .311; F (1,36) = 3.863; *p* = .057						
Constant	12.496			2.743	<.001		
Age	−.071	−.311		.036	.057	.311	1.000
Gait speed	R = .542; F (2,35) = 7.267; *p* = .002						
Const	−.580			.496	.250		
Steps	2.646E-5	.420		.009E-3	.005	.440	1.042
MNA	.052	.436		.017	.006	.453	1.042
TUG	R = .677; F (4,33) = 6.963; *p* < .001						
Const	21.983			4.843	<.001		
Age	−.026	−.098		.041	.525	−.111	1.409
MVPA	−.004	−.138		.009	.626	−.085	4.4778
Steps	−.166E-3	−.431		.109E-3	.138	−.256	4.890
MNA	−.417	−.575		.098	<.001	−.597	1.105
SPPB	R = .379; F (1,36) = 6.034; *p* = .019						
Const	6.358			2.076	.004		
MNA	.186	.379		.076	.019	.379	1.000

^a^ TUG, Timed Up and Go test; MVPA, moderate to vigorous physical activity; MNA, mini nutritional assessment; SE, Standardized Error; VIF, variation inflation factor.

The results indicated that the gait speed model explained 29.3% of the variance and was a significant predictor of gait speed F (2,35) = 7.267, *p* = .002. Both MNA and number of steps per day contributed significantly to the model (B = .052, *p* = .005; B = 2.646E-5, *p* = .006). The model for TUG explained 45.8% of the variance and was a significant predictor of TUG F (4,33) = 6.963, *p* = .001. Only MNA contributed significantly to the model (B = −.417, *p* < .001).

## 4 Discussion

The aim of this study was to determine the presence of sarcopenia and obesity parameters in selected active older adults and whether behavioural factors such as the amount of physical activity, sedentary behaviour, nutritional status, and Mediterranean lifestyle are associated with them. Our results show that three participants in our sample had sarcopenia (two of them were women), 18 of participants were obese (11 of them were women), and three of participants had sarcopenic obesity (two of them were women). Although we found a higher prevalence of obesity in women (61.1% of all obese participants were women), we found no differences between the selected behavioural factors (exercise and diet). Further analyses were therefore not performed separately by sex. The presence of sarcopenia is consistent with previously published literature about prevalence of sarcopenia (Keller, 2019; Smith et al., 2020; von Haehling et al., 2010). Interestingly, the percentage of obese individuals was relatively high despite having enrolled active older adults who reported being physically active on a regular basis. Despite the previously established association between a higher percentage of body fat and lower perceived quality of life in older adults (Giovannini et al., 2019), our participants reported an active, healthy lifestyle, as already reported in published study (Teraž et al., 2022). Because the definition of sarcopenic obesity has not been precisely defined in recent years, the prevalence of sarcopenic obesity in the literature varies widely (from 2.1% to 12%) (Baumgartner, 2006; Stenholm et al., 2008; Bahat, 2022). The most commonly used definition in the past, that was published by Baumgartner (2006), states that a high amount of fat mass, typically expressed as percent body fat greater than 27% in men and 38% in women, and the skeletal muscle index (skeletal mass normalized by the square of the participant’s body height in meters) of less than −1 or −2 SD of a sex-specific mean of young reference group are classified as sarcopenic obesity. Other studies showed (e.g., Newman et al., 2005) that this definition can underestimate sarcopenia in overweight and obese participants. Moreover, there is a wide range of diagnostic tools, criteria, and cut-off values for sarcopenia, obesity and sarcopenic obesity, which can lead to inconsistent results and reporting of prevalence. This is also reflected in the wide range of reported prevalence of sarcopenic obesity, which ranges from 4.4% to 17% in men and 3% to 14% in women (Zamboni et al., 2008). ESPEN and EASO launched an initiative to adopt a definition of SO, which is the co-existence of obesity and sarcopenia (Donini et al., 2022), but the evaluation of individuals with suspected SO was performed on two levels, screening, and diagnosis. Eighteen participants (47.4%) who took part in our study had increased BMI [which is the first condition of screening (Donini et al., 2022)]. Furthermore, three of them had surrogate indicators of sarcopenia such as age above 70 years in combination with various clinical symptoms or health risk factors. In all three we confirmed the diagnosis of SO with skeletal muscle functional parameter tests. Interestingly, all included participants had obesity in addition to sarcopenia and were therefore recorded as having SO. Majority of obese participants were women (61.1%), two of them had SO, confirming observations previously noted by Oh et al. (2015) but no other studies (Batsis et al., 2015; Perna et al., 2017; Du et al., 2019). Because an official definition of sarcopenic obesity has been proposed in the year 2022, the actual prevalence of sarcopenic obesity must await studies that categorize sarcopenic obesity according to the same system. In this regard, it would be useful to review the studies already conducted to determine the prevalence of SO according to the new proposed definition.

Furthermore, we analysed the association between behavioural variables, namely, PA, SB, steps, nutritional status, and parameters that are typical for sarcopenic obesity. When we ran a multiple linear regression model, we found that the nutritional status was the only factor that made a significant contribution to the model of TUG, the model of SPPB and the model of Chair stand test. On the other hand, to the gait speed model contributed two factors, number of steps per day and nutritional status of the individual. Our participants walked an average of 8,916 ± 3,543 steps per day, which according to the literature, puts them in a active older population (Tudor-Locke et al., 2011). We already know that the high number of daily steps has advantages to the individual’s health (Tudor-Locke and Bassett, 2004; Tudor-Locke et al., 2011). Some studies suggest that nutritional status affects gait speed in older adults (White et al., 2012; Asp et al., 2017). As Mendes et al. (2018) previously stated, we also found that nutritional status positively influences individual gait speed. However, knowing that gait speed is a commonly used indicator of physical performance in older adults, this is an important finding that may contribute to the overall management of older adults.

Interestingly, we found no association between sedentary time and selected parameters of sarcopenia and obesity. Donini et al. (2022) established that a sedentary behaviour may play a relevant role in the incidence of sarcopenia and obesity. On average, our participants wore the accelerometer for 15.2 h per day (we can assume that this is the time they were awake on average per day), of which approximately 10.8 h per day were spent sitting, and that is more than recommended (Bull et al., 2020). Smith et al. (2020) have shown that older adults who have more than 4 h/day SB, are at higher risk for sarcopenia (one of the conditions for sarcopenic obesity). Moreover, it is already known, a higher amount of PA at low intensity and a lower proportion of SB can have a positive effect on BMI (Bann et al., 2015). Moreover, total PA with a higher proportion of MVPA may protect against sarcopenia and have a positive effect on body composition (Rosique-Esteban et al., 2019; Marcos-Pardo et al., 2020). However, to our surprise, none of the selected behavioural factors (physical activity or inactivity and eating habits) correlated with the fat percentage or body mass index of the participants (two factors used to determine SO). Despite adherence to recommended daily step count (>7,000), 47.4% of participants were obese. Again, this could be due to the prolonged sedentary time or the eating habits of the participants. Adherence to the Mediterranean lifestyle has been shown to have a positive effect on the defence against excess body mass (Mendez et al., 2006; Beunza et al., 2010) and thus on excess fat mass and BMI. Although our participants were well nourished (the mean of MNA was 27.4) and adherence to the Mediterranean lifestyle was high, the reasons for the relatively high percentage of fat mass could be inadequate dietary intake. The dietary questionnaires included in the study did not analyse individual macronutrients; MNA is designed especially to assess malnutrition (Vellas, Garry, and Guigoz, 1999) and it has already been established that it is not useful to predict sarcopenia (Lengelé et al., 2021). Although our participants scored high on the MNA, we were unable to assess the extent to which they consumed nutrients that contribute to weight gain and increased fat mass, such as fatty meats, processed meat, full -fat dairy products, baked products, soft drinks, baked products and canned and processed foods (Shlisky et al., 2017). All mentioned leads to another possible explanation, namely, excessive energy intake, as a positive energy balance also influences obesity (Trouwborst et al., 2018).

Although regular, adequate physical activity and a reduction in sedentary behaviour are of high importance for health, problems such as obesity, sarcopenia, and sarcopenic obesity need to be addressed in more complex ways. The important strength of our study is objectively measured PA and SB on active older adults, who are underrepresented in the literature. Moreover, our findings suggest that the recommended level of physical activity for older adults may not be sufficient to counteract obesity and sarcopenic obesity and that the problem is more complex. These conclusions warrant further investigation because sarcopenia, obesity, and sarcopenic obesity are all significant health problems.

### 4.1 Limitations of the study

Some limitations should be addressed. The prevalence of sarcopenia and sarcopenic obesity determined in this study is comparable to other published studies. However, the “true” prevalence of sarcopenic obesity depends on the definition that used. The use of a standardized definition will provide a more realistic picture of the prevalence of sarcopenic obesity in future publications. Second, the sample is too small to draw firm conclusions, but it provides an indication of what needs to be explored further. Further insight into the dietary habits of active older people would be needed to assess what types of foods predominate in the diet of the selected sample and may be influencing the increased fat mass.

## Data Availability

The raw data presented in this study will be available on request from the corresponding author, without undue reservation.
